# Changes in ovarian morphology and hormone concentrations associated with reproductive seasonality in wild large Japanese field mice (*Apodemus speciosus*)

**DOI:** 10.1590/1984-3143-AR2021-0067

**Published:** 2021-12-24

**Authors:** Kazuki Komatsu, Kohsuke Murata, Tsugumi Iwasaki, Syun Tokita, Shiina Yonekura, Satoshi Sugimura, Yohei Fujishima, Akifumi Nakata, Tomisato Miura, Hideaki Yamashiro

**Affiliations:** 1 Graduate School of Science and Technology Niigata University Niigata Japan Graduate School of Science and Technology, Niigata University, Niigata, Japan; 2 Institute of Agriculture Tokyo University of Agriculture and Technology Tokyo Japan Institute of Agriculture, Tokyo University of Agriculture and Technology, Tokyo, Japan; 3 Department of Risk Analysis and Biodosimetry Institute of Radiation Emergency Medicine Hirosaki University Aomori Japan Department of Risk Analysis and Biodosimetry, Institute of Radiation Emergency Medicine, Hirosaki University, Aomori, Japan; 4 Department of Life Science Faculty of Pharmaceutical Sciences Hokkaido University of Science Sapporo Japan Department of Life Science, Faculty of Pharmaceutical Sciences, Hokkaido University of Science, Sapporo, Japan

**Keywords:** folliculogenesis, estradiol, oogenesis, seasonality, wild mice

## Abstract

Wild large Japanese field mice (*Apodemus speciosus*) responses to cyclic seasonal changes are associated with physiological and behavioral changes. However, the detailed regulation of oogenesis in the ovary during the seasonal reproductive cycle in wild large Japanese field mice has not been studied. We assessed the dynamics and changes in ovarian morphology and hormone concentrations associated with reproductive seasonality throughout the year. The stages of the ovarian morphological breeding cycle of wild large Japanese field mice were classified as breeding, transition, and non-breeding periods during the annual reproductive cycle. Measurement of blood estradiol concentrations throughout the year showed that the levels in September and October were higher than those in other months. It is presumed that follicle development starts from a blood estradiol concentration of 38.4 ± 27.1 pg/mL, which marks a shift from the transitional season to the breeding season, followed by the transition to the non-breeding season at 26.1 ± 11.6 pg/mL. These results suggest that seasonal follicle development in wild rodents is correlated with estradiol regulation. We consider this species to be an alternative animal model for studying seasonal reproductive changes and the effects of environmental changes.

## Introduction

Many mammals are seasonal breeders and respond to seasonal changes by adjusting their physiology and behavior (Fuentes et al., 1991). Seasonal reproduction is a common adaptive strategy among mammals that allows breeding to occur at times of the year when it is most advantageous for the subsequent survival and growth of offspring. A major mechanism responsible for seasonal reproduction is a remarkable increase in the responsiveness of gonadotropin-releasing hormone (GnRH) neurons to the negative feedback effects of estradiol (Weems et al., 2015). The switching on and off of reproductive function during the annual breeding cycle is the most striking example of spermatogenesis- and/or oogenesis-induced processes. However, detailed studies on seasonal reproductive changes in wild animals have severe limitations, as wild animals are not easily captured and/or kept in captivity (Lincoln and Short, 1980). Therefore, it is of interest to identify a more suitable experimental animal that predominantly has seasonal reproductive activity, is small and tractable enough to permit repeated sampling, and has minimal maintenance requirements.

Our initial interest in seasonal reproductive changes was aroused by studies of large Japanese field mice (*Apodemus speciosus*) living in forests throughout Japan. We consider this species to be an excellent animal model for studying spermatogenesis, oogenesis, and the effects of environmental changes on these processes (Takino et al., 2017). We have previously reported on the nature of testicular regression and spermatogenic arrest in the species throughout the year (Akiyama et al., 2015). Recently, we have shown that spermatogenesis during the seasonal reproductive cycle is controlled by the proliferation and apoptosis of germ cells in this species (Ito et al., 2020). Furthermore, we successfully established a protocol for collecting ovulated oocytes from wild large Japanese field mice by administering inhibin antiserum and equine chorionic gonadotropin during both the reproductive and non-reproductive seasons (Meguro et al., 2019). However, the detailed regulation of oogenesis in the ovary during the seasonal reproductive cycle in wild large Japanese field mice has not been studied.

This study aimed to characterize the dynamics and changes in ovarian morphology and hormone concentrations in wild large Japanese field mice associated with reproductive seasonality throughout the year.

## Materials and methods

### Collection of wild large Japanese field mice

The principles of laboratory animal care were followed during this study, and all procedures were conducted in accordance with the guidelines provided by the Ethics Committee for Care and Use of Laboratory Animals for Research of the Niigata University, Japan (Protocol No. 26-80-2). The capture of undomesticated rodents in the sampling area was permitted by the Niigata City Office.

Wild large Japanese field mice were captured from April 2017 to December 2018 at Kakuta Mountain in Niigata, Japan. Individual wild large Japanese field mice were captured using Sherman-type live traps baited with peanuts, as described previously (Akiyama et al., 2015). Female wild large Japanese field mice (*n* = 45) were used in this experiment (Table 1). The mice were sacrificed by cervical dislocation on the day of capture. The ovaries were isolated from each wild mice (*n* = 22), and one ovary from each mice was fixed in Bouin’s solution. Individual serum samples were collected and stored at −80 °C (*n* = 44). Wild mice were included in both analyses. Efforts were made to minimize the number of wild mice captured based on the 3Rs principle (reduction, refinement, and replacement) in animal experiments.

**Table 1:** Individual body weight and ovary size of the large Japanese field mice.

**Mice No.**	**Sampling date**	**Body weight (g)**	**Ovary length (mm)**	**Ovary weight (g)**	**HE staining**	**ELISA analysis**
1	4/5/2017	24.1	2.0	0.0022	---	Done
2	4/6/2017	29.0	3.0	0.0043	---	Done
3	4/13/2017	31.2	1.5	0.0016	Done	Done
4	4/20/2017	22.4	1.5	0.0043	--	Done
5	4/28/2017	49.5	2.5	0.0045	Done	Done
6*	4/28/2017	44.7	2.0	0.0058	Done	Done
7	5/11/2017	33.4	1.5	0.0027	Done	Done
8	5/11/2017	25.2	2.0	0.0044	Done	Done
9	6/15/2017	39.0	2.0	0.0012	---	Done
10	6/29/2017	32.4	2.0	0.0026	Done	---
11	6/29/ 2017	33.4	2.0	0.0008	Done	Done
12	7/5/2017	41.2	2.1	0.0031	Done	Done
13	7/11/ 2017	40.7	1.7	0.003	---	Done
14	7/11/2017	27.5	1.6	0.002	---	Done
15	7/14/2017	38.1	1.8	0.0015	---	Done
16	8/25/2017	27.6	2.7	0.0033	Done	Done
17	8/29/ 2017	32.2	3.0	0.0021	Done	Done
18	8/29/2017	44.6	3.0	0.0035	---	Done
19	9/20/2017	31.3	3.0	0.0049	Done	Done
20	9/26/2017	34.7	2.5	0.0069	---	Done
21	9/29/2017	37.3	2.5	0.0046	Done	Done
22	9/29/2017	31.6	4.0	0.0075	---	Done
23	10/26/2017	31.3	1.5	0.0012	Done	Done
24	10/27/ 2017	31.3	3.0	0.0018	---	Done
25	10/31/ 2017	35.6	3.0	0.0086	Done	Done
26	11/27/ 2017	37.3	---	---	---	Done
27	11/27/2017	30.7	1.5	0.0009	Done	Done
28	11/30/2017	29.1	3.1	0.0021	---	Done
29	12/15/2017	21.6	2.2	0.0015	Done	Done
30	12/15/2017	24.9	1.0	0.0012	Done	Done
31	12/21/2017	25.2	1.8	0.001	Done	Done
32	12/21/2017	29.1	2.0	0.0017	---	Done
33	1/30/2018	27.1	2.0	0.0019	Done	Done
34	2/28/2018	22.1	2.0	0.0039	Done	Done
35	2/28/2018	30.3	2.2	0.0068	Done	Done
36*	3/20/2018	32.5	2.8	0.002	Done	Done
37	4/17/2018	31.0	3.0	0.0043	---	Done
38	5/22/2018	35.6	2.0	0.003	---	Done
39	5/22/ 2018	36.3	2.0	0.0013	---	Done
40	6/20/2018	34.7	1.0	0.0008	---	Done
41	10/4/2018	35.1	2.3	0.0069	---	Done
42	10/4/2018	45.6	2.5	0.0056	---	Done
43	10/17/2018	31.5	---	---	---	Done
44	11/27/2018	34.0	---	---	---	Done
45	12/5/2018	24.9	1.8	0.0008	---	Done

* No.6 and 36 were pregnant.

### Morphological assessment of ovaries for classification of stages of folliculogenesis

Individual ovaries (*n* = 22) were fixed in Bouin’s solution. Fixed ovaries were embedded in paraffin and stained using hematoxylin and eosin (HE), according to a previously described method (Ito et al., 2020; Komatsu et al., 2021). The ovaries were then briefly dehydrated in a series of different concentrations of alcohol, made transparent by treatment with toluene, embedded in paraffin, and cut into 4 μm-thick sections before staining. Folliculogenesis was evaluated as the mean value of 20 serial follicle sections following modified Pedersen’s follicular classification (Pedersen and Peters, 1968; Pedersen, 1970): small (primordial or primary follicles), medium (prenatal follicles), large (antral or Graafian follicles), and corpora lutea (CL) (Yoshida et al., 2009). The samples were further subdivided into five types based on their morphological appearance and follicle size in Table 2 and classified in Table 3. Large follicles without oocytes were counted as antral or Graafian follicles. CL was defined as one estrus cycle after ovulation during the reproductive season.

**Table 2:** Follicle classification and corpora lutea in wild large Japanese field mice.

**Follicles**	**Synonym**	**Characteristics**	**Size**
Small	Type 1	Primordial follicles with few granulosa cells	10-20 µm
Medium	Type 2	Primary follicles with one granulosa cell layer surrounding the oocyte	20-150 µm
	Type 3	Secondary follicles with more than two granulosa cell layers surrounding a growing oocyte	150-250 µm
Large	Type 4	Pre-antral follicles with many granulosa cell layers and scattered fluid areas surrounding a large oocyte	250-400 µm
	Type 5	Large antral or Graafian follicles with a single cavity, follicle fluid, and well-formed cumulus cells	400-500 µm
Corpora lutea	CL	After a follicle ovulates, a transient endocrine structure form	

The number of atretic follicles was not determined.

**Table 3:** Number of each follicles type and corpora lutea throughout the year in ovary of the wild large Japanese field mice.

**Month**	**Total No. of wild mice**	**Total No. of Follicle**	**Small**	**Medium**		**Large**		**Corpora lutea**
			**Type 1**	**Type 2**	**Type 3**	**Type 4**	**Type 5**	
April	3	11.3 ± 2.5	7.3 ± 2.1	2.0 ± 0.8	1.7 ± 1.7	0 ± 0	0.3 ± 0.5	0.3 ± 0.5
May	2	10.5 ± 0.5	3.5 ± 0.5	2.5 ± 1.5	4.0 ± 0.0	2.5 ± 1.5	0 ± 0	0 ± 0
June	2	11.0 ± 5.0	4.0 ± 2.0	3.5 ± 1.5	2.0 ± 0.0	2.0 ± 1.0	0 ± 0	0.5 ± 0.5
July	1	15.0	1.0	5.0	3.0	3.0	3.0	0
August	2	14.5 ± 3.5	5.5 ± 0.5	4.0 ± 1.0	1.5 ± 0.5	2.5 ± 1.5	1.0 ± 1.0	0 ± 0
September	2	28.0 ± 4.0	6.0 ± 1.0	7.5 ± 0.5	6.0 ± 1.0	1.0 ± 0.0	2.5 ± 1.5	0.5 ± 0.5
October	2	17.0 ± 4.0	4.0 ± 1.0	6.0 ± 1.0	4.0 ± 2.0	3.0 ± 2.0	1.5 ± 0.5	0 ± 0
November	1	17.0	9.0	5.0	3.0	0	0	0
December	3	11.3 ± 3.7	3.3 ± 2.1	4.7 ± 0.5	3.0 ± 1.4	0.3 ± 0.5	0 ± 0	0 ± 0
January	1	12.0	3.0	4.0	3.0	2.0	0	0
February	2	18.5 ± 0.5	6.0 ± 2.0	3.0 ± 2.0	3.0 ± 0.0	3.0 ± 0.0	3.5 ± 0.5	1.0 ± 1.0
March	1	12.0	4.0	2.0	2.0	1.0	3.0	1.0

The reproductive cycle of the Japanese field mice was classified as follows based on the follicle classification of the ovaries: breeding period (Types 1 to 5, except April), Transition period (Types 1 to 4), and non-breeding period (Types 1 to 3). The size of corpora lutea is not indicated. Values are presented as mean ± SD.

### Measurement of estradiol in blood

Estradiol concentration was measured using a commercial enzyme-linked immunosorbent assay (ELISA) kit (No. 582251, Cayman Chemical, MI, USA) following the manufacturer’s protocol. Individual serum samples (110 µL, *n* = 44) were mixed with diethyl ether (330 µL) to extract estradiol and incubated for 1 h. After freezing in liquid nitrogen, the liquid phase was decanted into a 10 mL test tube and completely volatilized until odorless through bathing in a 50 °C water bath in a draft. After volatilization, the assay buffer (110 µL) was added to the test tube. The sample was dispensed into 50 µL duplicates, ‘Estradiol AChE Tracer’ and ‘Estradiol ELISA Antiserum’ were added to the samples, and the samples were incubated for 1 h. After washing five times with washing buffer, Ellman’s reagent was added to the samples and they were incubated for 1 h. The optical absorbance was measured at 412 nm. The estradiol concentration was calculated using an estradiol standard curve.

### Statistical analysis

**Figure 1:**
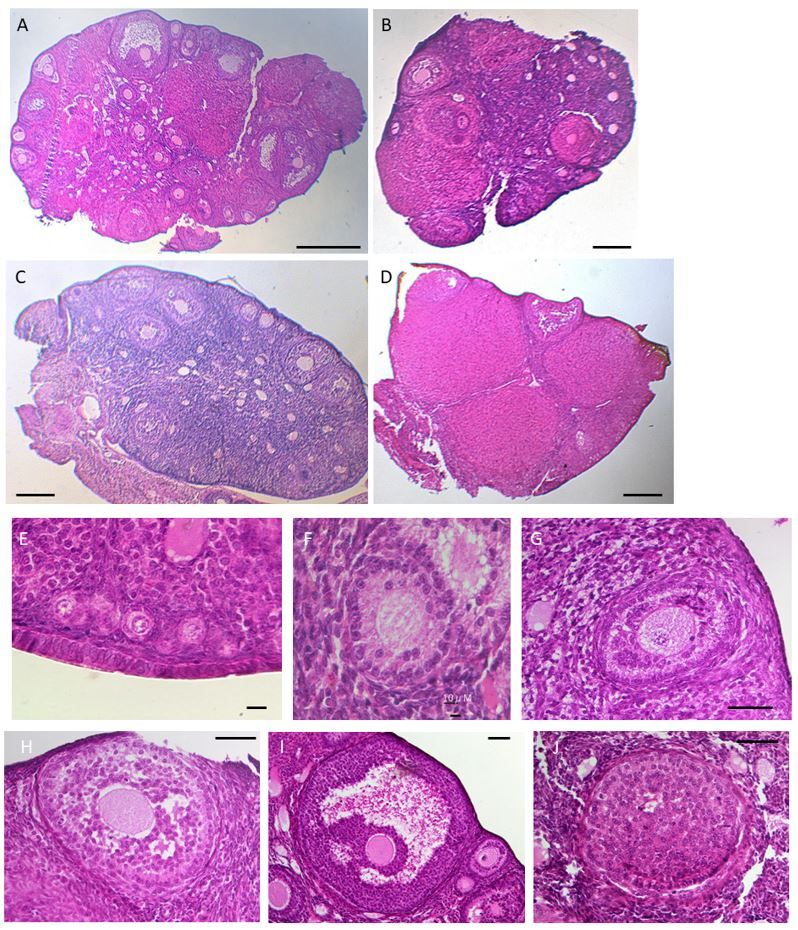
Histological seasonal changes of the ovary and classification of the stages of folliculogenesis; Photomicrographs of haematoxylin-eosin stained sections of wild large Japanese field mice ovary, A, Breeding period; B, Transition period; C; Non-breeding period; D, Corpora lutea, scale bar = 200 μm. E, Type 1; primordial follicles with few granulosa cells, scale bar = 10 μm. F, Type 2; Primary follicles with one granulosa cell layer surrounding the oocyte, scale bar = 10 μm. G, Type 3; Secondary follicles with more than two granulosa cell layers surrounding a growing oocyte, scale bar = 100 μm. H, Type 4; Pre-antral follicles with many granulosa cell layers and scattered fluid areas surrounding a large oocyte, scale bar = 100 μm. I, Type 5; Large antral or Graafian follicles with a single cavity, follicle fluid, and well-formed cumulus cells, scale bar = 100 μm. J, Corpora lutea; After a follicle ovulates, a transient endocrine structure forms, scale bar = 100 μm.

**Figure 2:**
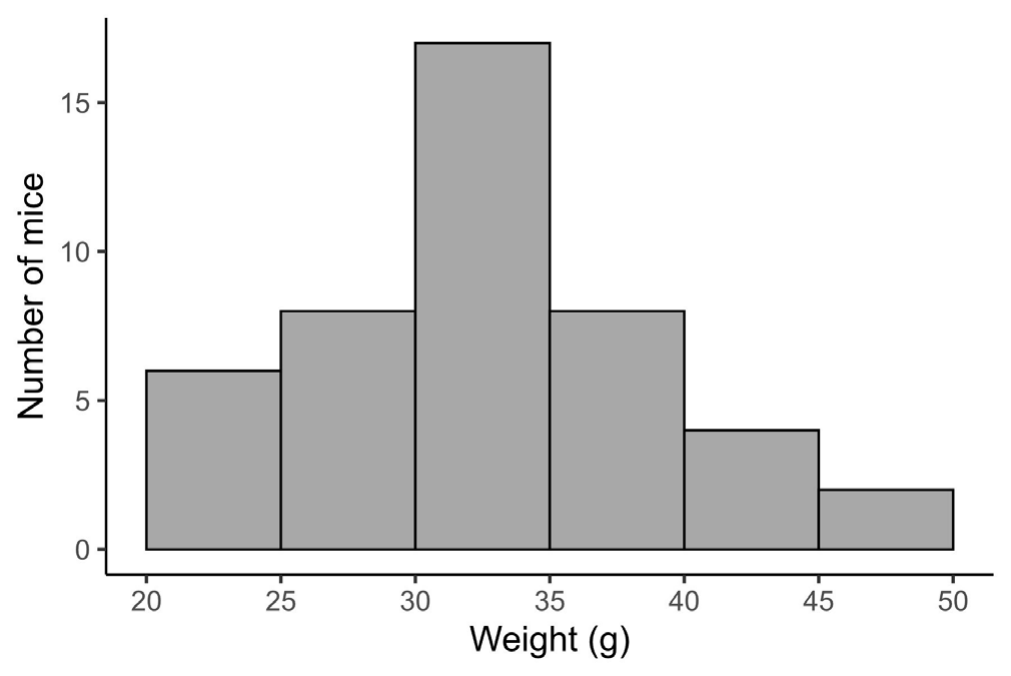
Histogram of the wild large Japanese field mice number by body weight.

**Figure 3:**
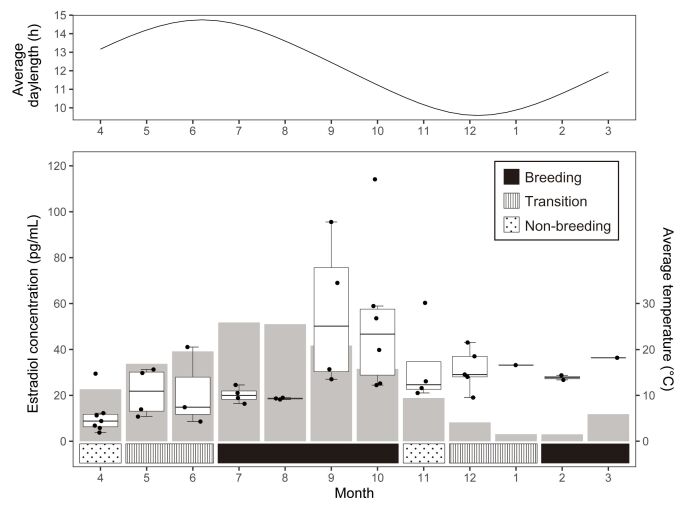
ELISA analysis of blood estradiol concentration per month (box plot); temperature (bar plot) and photoperiod hours in the sampling region during the execution of the experiment.

**Figure 4:**
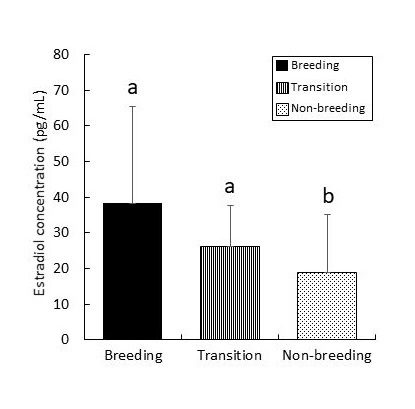
ELISA analysis of blood estradiol concentration in wild large Japanese field mice during the seasonal reproductive cycle. Values are represented as mean ± SD. ^a, b^ Different superscripts denote significant differences (*p* < 0.05).

**Figure 5:**
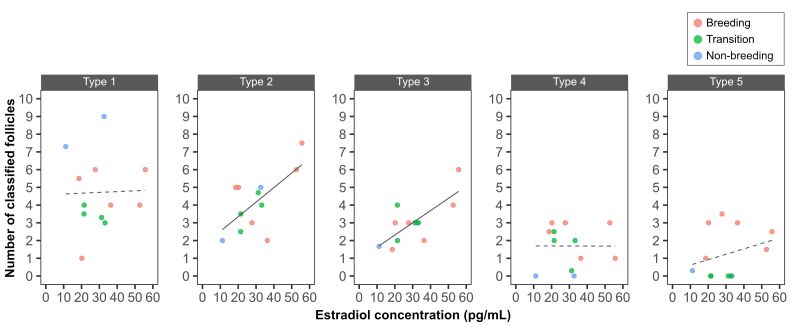
Correlation between the concentration of estradiol in the blood and the number of classified follicles.

Statistical analyses were performed using STATVIEW Version 5.0 (Abacus Concepts Inc., Berkeley, CA, USA). Figures 1 to 3 were not performed statistical analyses. Data were classified according to breeding seasons, and differences between the groups were analyzed using one-way analysis of variance (ANOVA) with Fisher’s protected least-significant difference post-hoc test (Table4 and Figure 4). Data are expressed as mean ± SD. Statistical significance was set at *p* < 0.05. The correlation coefficients were obtained using a simple regression analysis (Figure 5).

**Table 4:** Number of each follicles type during reproductive periods in ovary of the wild large Japanese field mice.

**Reproductive period**	**Total No. of wild mice**	**Total No. of follicle**	**Small**	**Medium**	**Large**
			**Type 1**	**Type 2 and 3**	**Type 4 and 5**
Breeding	10	18.7 ± 5.8^a^	5.8 ± 3.0 (26.2)^a^	7.4 ± 3.6 (44.8)^a^	6.0 ± 2.6 (30.6)^a^
Transition	8	11.3 ± 3.7^a^	3.5 ± 1.8 (31.1)^a^	6.8 ± 1.8 (60.0)^b^	1.1 ± 1.0 (10.0)^b^
Non-breeding	4	12.8 ± 3.8^a^	7.8 ± 2.2 (60.8)^b^	4.8 ± 3.3 (37.3)^a^	0.3 ± 0.5 (2.0)^c^

Values are expressed as mean percentages ± SEM (%/total No. of follicles). ^a-c^ Different superscripts within the same column denote significant differences (*p* < 0.05).

## Results

### Histological analysis using HE staining

Figure 1 shows the HE-stained images of the follicles and ovaries of wild large Japanese field mice. After classifying follicles into five types according to the HE-stained images, it was observed that Type 1–3 and 5 follicles were present in the ovaries of mice caught in April (Table 3). In the ovaries of mice collected from May to June, 2.5 ± 1.5 and 2.0 ± 1.0 Type 4 follicles were observed and accounted for approximately 18–24% of the total number of follicles. In the ovaries collected from July to October, 6.9–20% of the total number of follicles were Type 5 follicles. The total number of follicles in September was higher than that in the other months. No Type 5 follicles were observed in the ovaries from November to January. In the ovaries of mice collected from December to January, up to Type 4 follicles were observed. In the ovaries of mice collected from February to March, the number of Type 5 follicles increased, accounting for approximately 19–25% of the total number of follicles. Consequently, the reproductive cycle of the Japanese field mice was classified as follows based on the follicle classification of the ovaries: breeding period (Types 1 to 5), Transition period (Types 1 to 4), and non-breeding period (Types 1 to 3). April was found to be a non-breeding period, May to June a transition period, July to October a breeding period, November a non-breeding period, December to January a transition period, and February to March a breeding period.

Type 1 follicles accounted for approximately 26.2%, 31.1%, and 60.8% of the total follicles in the breeding, transition, and non-breeding periods, respectively, and significant differences (*p* < 0.05) were observed between breeding, transition, and non-breeding periods (Table 4). Type 2 to Type 3 follicles accounted for 44.8%, 60.0%, and 37.3% of the total follicles in the breeding, transitional, and non-breeding seasons, respectively, and significant differences (*p* < 0.05) were observed between transition and breeding and non-breeding periods. Types 4 and 5 follicles accounted for approximately 30.6%, 10.0%, and 2.0% in the breeding, transitional, and non-breeding seasons, respectively, and there were significant differences (*p* < 0.05) between these three periods.

Body size of wild mice did not appear to correlate with breeding season classification (Figure 2).

### Estradiol concentration

The concentration of estradiol in the blood throughout the year was measured by ELISA and temperature and photoperiod hours in the sampling region were showed during the execution of the experiment. With respect to the seasonal dynamics of the year, it remained at the lowest values between April and August, while September and October showed the highest values (Figure 3). Intermediate blood estradiol levels were observed between November and March.

The concentration of estradiol in the blood was 38.4 ± 27.1 pg/mL during the breeding period, which was significantly (*p* < 0.05) higher than the concentration observed (19.0 ± 16.3 pg/mL) in the non-breeding periods (Figure 4). There was no significant difference between the estradiol concentration in the blood between the breeding and transition periods.

Figure 5 shows the correlation between different months with respect to the concentration of estradiol in the blood. No strong correlation was observed between the concentration of estradiol in the blood and any follicle type over the year. However, a positive correlation was observed between estradiol concentration in the blood and Type 2 (*r* = 0.656) and Type 3 (*r* = 0.741) follicles.

## Discussion

The ovarian activity of female wild large Japanese field mice was classified by assessing changes in folliculogenesis. The ovarian follicular population showed different patterns throughout the year: low, medium, and high follicular populations were observed in three different reproductive seasons. In other words, two breeding, non-breeding, and transitional periods were observed during the annual reproductive cycle. These resulted in approximately the same breeding cycle and timing of breeding, transitional, and non-breeding periods as those found in male wild large Japanese field mice. In addition, blood estradiol concentration was found to be highest during the period from September to October, indicating that these months were the most favorable for breeding. This could be attributed to the activation of reproductive physiology or increased hormonal sensitivity during these months compared with that during the breeding season in September and October, or it may be due to environmental factors such as light, temperature, and food.

We next focused on Type 1, Type 2, and Type 3 during each reproductive period. At a blood estradiol concentration of approximately 19.0 ± 16.3 pg/mL, the percentage of Type 1 follicles was higher than the percentages of Type 2 and 3, and at 26.1 ± 11.6 pg/mL, the percentages of Type 2 and 3 follicles was higher than the percentage of Type 1. As a result, it was presumed that follicle development starts from a blood estradiol concentration of 26.1 ± 11.6 pg/mL, which induces the shift from the non-breeding season to the transitional season, followed by the transition to the breeding season at 38.4 ± 27.1 pg/mL. Comparing the changes in blood estradiol levels with the morphological dynamics of the ovaries and the reproductive cycle over the year revealed different periods of the annual breeding cycle.

Sheep show distinct annual patterns of reproductive activity in females, which are comprised of periods of ovarian activity and ovulation, termed the breeding season, which alternate with periods of ovarian quiescence and anovulation, referred to as anestrus (Lincoln and Short, 1980). These seasonal variations in reproductive activity at the ovarian level are the direct result of changes in the brain and result from two complementary alterations in the control of hypothalamic function, particularly in the regulation of GnRH secretion (Weems et al., 2015). In these species, GnRH pulses are controlled by the negative feedback actions of both estradiol and progesterone, whereas the GnRH surge is triggered by the positive feedback action of high estradiol concentrations at the end of the follicular phase of the ovarian cycle (Nestor et al., 2018). Therefore, there are more antral follicles during anestrus than during the peak of the breeding season due to increases in serum estrogen concentrations (Barrett et al., 2004; McNatty et al., 1984). However, sheep show similar changes in ovarian morphology and seasonal changes in blood estradiol levels during the year; that is, blood estradiol levels also increase as follicles develop in the fall of the breeding season (Arens et al., 2007; McNatty et al., 1984). Follicular development and changes in blood estradiol levels are minimal during non-breeding periods.

Conversely, the ovarian morphology of the female wild large Japanese field mice indicated a breeding season in July and August, although the blood estradiol concentration during these months was low. As a result, July and August could be thought of as the preparatory breeding period, where the ovarian morphology is in the reproductive period, but the blood estradiol concentration is yet to increase. However, male wild large Japanese field mice enter their breeding season during the preparatory breeding period of females. There are several possible reasons for these dynamics in July and August. Females have a gestation period and a child-rearing period, so the maximum number of offspring is limited. We considered that female wild large Japanese field mice increased their chances of mating by bringing their body to a fertile state faster than males and adjusting their physiological activity according to the estrus of males. In addition, the female transition period is longer than the male transition period; this transition from the non-breeding period to the breeding period may take longer owing to differences in genital structure. Because this has not been reported thus far in other seasonal breeding animals, it may be a useful bioresource for elucidating the mechanism of seasonal change in the breeding cycle.

Laboratory mice are known to be annual breeders. It has been reported that blood estradiol concentration in the estrus cycle increases to a maximum in the proestrus period, decreases in the estrus period, and decreases to a minimum in the metoestrus period, with concentration in the anestrus period in the range of about 3–7 pg/mL (Schwartz et al., 2004). The blood estradiol concentration in the reproductive cycle of female wild large Japanese field mice and in the estrus cycle of laboratory mice were compared. The concentration in wild large Japanese field mice varies in the range of 3.8–114.1 pg/mL. The maximum and average secretions differed greatly between the two groups. Although laboratory mice generally live in a constant environment, wild large Japanese field mice experience changes in their environmental conditions, such as light, temperature, and food, throughout the year. Therefore, it is considered that the range of estradiol secretion is increased and regulated so that it can adapt to these environments and reproduce.

## Conclusion

In summary, we characterized the breeding cycle of female wild large Japanese field mice. We found that there is a contradiction between follicle development and blood estradiol concentration and a difference in follicle development in laboratory mice. We consider this species to be an alternative animal model for studying seasonal reproductive changes and the effects of environmental changes.
